# Objective Outcome Evaluation of the Project P.A.T.H.S. in Hong Kong: Findings Based on Individual Growth Curve Models

**DOI:** 10.1100/tsw.2010.18

**Published:** 2010-02-12

**Authors:** Daniel T.L. Shek

**Affiliations:** ^1^ Department of Applied Social Sciences, The Hong Kong Polytechnic University, Shanghai, China; ^2^ Department of Sociology, East China Normal University, Shanghai, China; ^3^ Kiang Wu Nursing College of Macau, Macao

**Keywords:** adolescents, positive youth development, randomized group trial, Project P.A.T.H.S., objective outcome evaluation

## Abstract

The Tier 1 Program of the Project P.A.T.H.S. (Positive Adolescent Training through Holistic Social Programmes) is a curricular-based program that attempts to promote positive youth development in Hong Kong. In the second year of the Full Implementation Phase, 20 experimental schools (n = 2,784 students) and 23 control schools (n = 3,401 students) participated in a randomized group trial. Analyses based on linear mixed models via SPSS showed that participants in the experimental schools displayed better positive youth development than did participants in the control schools based on different indicators derived from the Chinese Positive Youth Development Scale. Differences between experimental and control participants were also found when students who joined the Tier 1 Program and perceived the program to be beneficial were employed as participants of the experimental schools.

## INTRODUCTION

The Project P.A.T.H.S. (**P**ositive **A**dolescent **T**raining through **H**olistic **S**ocial Programmes) is a youth enhancement program that attempts to promote holistic youth development in Hong Kong[1]. There are two tiers of programs (Tier 1 and Tier 2) in this project. The Tier 1 Program is a universal positive youth development program based on 15 positive youth development constructs[2] in which students in Secondary 1 to 3 take part. Since the inception of the Project, different evaluation strategies have been employed to evaluate the project[3,4,5]. Generally speaking, different stakeholders had positive perceptions of the program and there is support for the effectiveness of the program.

As far as objective outcome evaluation is concerned, several studies have shown that students who participated in the project showed better development than did the control participants. Utilizing a pre-experimental design, Shek[6] collected pre- and post-test data, utilizing the Chinese Positive Youth Development Scale (CPYDS), from 546 students participating in the 20-h Tier 1 Program of the Project P.A.T.H.S. Results showed that there were positive changes in the program participants in many measures of positive youth development. Although there was some increase in problem behavior in some areas, adolescent problem behavior was generally stable.

Based on the first two waves of data collected in a randomized group trial, Shek et al.[7] carried out analyses of covariance and linear mixed models, controlling for differences between the experimental and control groups in terms of pretest scores, personal variables, and random effects of schools. Results showed that participants in the experimental schools had significantly higher positive youth development levels than did participants in the control schools at post-test based on different indicators derived from the CPYDS. Based on the first four waves of data collected in the first 2 years of the Full Implementation Phase, Shek[8] found similar results by using analyses of covariance. In addition, the students in the experimental schools also displayed a lower level of delinquency, but better school adjustment than did students in the control schools.

Although the above findings provide support for the effectiveness of the Tier 1 Program of the Project P.A.T.H.S., it is noteworthy that ANOVAs and ANCOVAs (i.e., general linear models) were mainly used in the related studies. Besides the conventional methods based on MANCOVAs (e.g., [9]), some advanced techniques, including hierarchical linear modeling and latent growth curve modeling, have been developed in the past few decades[10,11]. In their review of different strategies for analyzing longitudinal data, Bijleveld et al.[12] reviewed nine methods to analyze longitudinal data. Hser et al.[13] proposed several methods of analyzing long-term treatment effects, including structural equation model, autoregressive panel model, multilevel/hierarchical linear model, latent growth curve model, survival/event history analysis, Markov model/(latent) transition model, and time-series analysis. While some of these models are compatible with each other, some of them are not. For example, there are studies that attempt to compare the multilevel model and latent growth curve model[14,15]. Among these strategies, hierarchical linear modeling is a strategy that is commonly used by researchers to examine changes in the program participants over time. In the present analyses, hierarchical linear modeling based on SPSS (Statistical Package for Social Sciences) was used primarily to examine the treatment effects over time.

## METHODS

### Participants and Procedures

Shek and associates[7] described the procedures and criteria for recruiting the initial 24 experimental schools (one school dropped out after Wave 1 and three schools withdrew after Wave 2) and 24 control schools in Year 1, during which the Waves 1 and 2 data were collected from Secondary 1 students. In Year 2, Waves 3 and 4 data were collected from the same cohort promoted to Secondary 2, with 20 experimental schools and 23 control schools. The number of students who joined the experimental group and control group in Years 1 and 2, and the number of completed questionnaires collected can be seen in Table 1.

At pre- and post-test, the purpose of the study was mentioned, and confidentiality of the data collected was repeatedly emphasized to all students in attendance on the day of testing. Parental and student consent was obtained prior to data collection. All participants responded to all scales in the questionnaire in a self-administration format. Adequate time was provided for the participants to complete the questionnaire. A trained research assistant was present throughout the administration process.

### Instruments

Consistent with the procedures used in Year 1, the participants were invited to respond to a questionnaire that comprised different measures of youth development at pretest (i.e., before the program began) and post-test (i.e., after the program ended). The following measures were used.

**TABLE 1**
**Number of Participants and Completed Questionnaires Collected at Year 1 (Waves 1 and 2) and Year 2 (Waves 3 and 4)**

| Cases | Year 1 | | | Year 2 | | |
|---|---|---|---|---|---|---|
| | Experimental | Control | Total | Experimental | Control | Total |
| Pretest questionnaire collected | 4,121 | 3,854 | 7,975 | 3,290 | 3,861 | 7,151 |
| Pretest questionnaire available for matching | 4,050 | 3,795 | 7,845 | 3,276 | 3,845 | 7,121 |
| Post-test questionnaire collected | 3,914 | 3,770 | 7,684 | 3,047 | 3,764 | 6,811 |
| Post-test questionnaire available for matching | 3,880 | 3,728 | 7,608 | 3,047 | 3,763 | 6,810 |
| Successfully matched | 3,312 (49.6%) | 3,363 (50.4%) | 6,675 (100%) | 2,784 (45.0%) | 3,401 (55.0%) | 6,185 (100%) |

*Note*: The number (percentage) of the successfully matched cases across Waves 1–4 is 5,054 (100%); experimental group: 2,236 (44.2%); control group: 2,818 (55.8%).

## *Chinese Positive Youth Development Scale (CPYDS)*

Based on the analyses conducted in Year 1, the item composition of the 15 subscales of the CPYDS is listed as follows (pretest refers to the Wave 1 data and post-test refers to the Wave 4 data):

1. Bonding Subscale (six items): $\alpha$ = 0.86 and 0.88 at pre- and post-test.
2. Resilience Subscale (six items): $\alpha$ = 0.88 and 0.88 at pre- and post-test.
3. Social Competence Subscale (seven items): $\alpha$ = 0.87 and 0.87 at pre- and post-test.
4. Emotional Competence Subscale (six items): $\alpha$ = 0.86 and 0.86 at pre- and post-test.
5. Cognitive Competence Subscale (six items): $\alpha$ = 0.87 and 0.88 at pre- and post-test.
6. Behavioral Competence Subscale (modified five items): $\alpha$ = 0.82 and 0.82 at pre- and post-test.
7. Moral Competence Subscale (six items): $\alpha$ = 0.81 and 0.80 at pre- and post-test.
8. Self-Determination Subscale (five items): $\alpha$ = 0.82 and 0.81 at pre- and post-test.
9. Self-Efficacy Subscale (modified two items): $\alpha$ = 0.58 and 0.59 at pre- and post-test.
10. Beliefs in the Future Subscale (modified three items): $\alpha$ = 0.84 and 0.84 at pre- and post-test.
11. Clear and Positive Identity Subscale (seven items): $\alpha$ = 0.87 and 0.86 at pre- and post-test.
12. Spirituality Subscale (seven items): $\alpha$ = 0.91 and 0.91 at pre- and post-test.
13. Prosocial Involvement Subscale (five items): $\alpha$ = 0.86 and 0.85 at pre- and post-test.
14. Prosocial Norms Subscale (five items): $\alpha$ = 0.81 and 0.81 at pre- and post-test.
15. Recognition for Positive Behavior Subscale (four items): $\alpha$ = 0.83 and 0.83 at pre- and post-test.

As mentioned by Shek[8], different composite indices derived from the scale were used to assess positive youth development. In this paper, findings based on several measures were tentatively reported and the findings based on a fuller range of measures will be reported elsewhere. First and foremost, according to Shek et al.[7], the mean of the total mean scores based on 12 subscales (excluding behavioral competence, self-determination, and prosocial norms) could be used as an overall measure of positive youth development (CPYDS-12: $\alpha$ = 0.94 and 0.93 at pre- and post-test). Next, as it can be argued that constructs including spirituality, prosocial norms, prosocial involvement, bonding, and recognition for positive behavior are different from the rest of the scale, a summation of 10 subscales (CPYDS-10: $\alpha$ =

0.94 and 0.94 at pre- and post-test) assessing psychosocial competence and strengths was used (i.e., resilience, social competence, emotional competence, cognitive competence, behavioral competence, moral competence, self-determination, self-efficacy, beliefs in the future, and clear and positive identity). Third, based on conceptual analyses of the items, one key item was derived for each domain, which resulted in a 15-item key measure (KEY15: $\alpha$ = 0.90 and 0.90 at pre- and post-test). Fourth, based on item analysis, a 36-item key measure was derived (KEY36: $\alpha$ = 0.96 and 0.96 at pre- and post-test). Shek and Ma[16] also showed that the 15 subscales in the CPYDS could be further reduced to four dimensions, including cognitive-behavioral competencies, prosocial attributes, positive identity, and general positive youth development qualities.

### *School Adjustment Measures (SA)*

Three items were used to assess the school adjustment of the participants. The first item assessed a respondent's perception of his or her academic performance when compared with schoolmates in the same grade. The respondents were asked to rate "Best", "Better than usual", "Ordinary", "Worse than usual", or "Worst" in this item. The second item assessed the respondent's satisfaction with his or her academic performance. The respondents were asked to rate "Very satisfied", "Satisfied", "Average", "Dissatisfied", or "Very dissatisfied" in this item. The final item assessed the respondent's perception of his or her conduct. The respondents were asked to rate "Very good", "Good", "Average", "Poor", or "Very poor" in this item. Previous research findings showed that these three items and the related scale were temporally stable and valid[9]. The present findings showed that the measure was internally consistent at pretest ($\alpha$ = 0.72) and post-test ($\alpha$ = 0.73) in Year 2.

### *Subjective Outcomes Scale (SOS)*

Twenty items were used to assess the participant's satisfaction with the program and instructor, as well as their perceived benefits of the program at post-test (i.e., Wave 2 data). The response options included "Strongly disagree", "Moderately disagree", "Slightly disagree", "Slightly agree", "Moderately agree" and "Strongly agree". Reliability analysis showed that this measure was reliable ($\alpha$ = 0.97). Item 20 of this scale is "Overall speaking, the program was beneficial to my development".

## Data Analytic Strategies

The data were analyzed by linear mixed models (LMM) via SPSS with maximum likelihood estimation[17,18,19]. Basically, individual growth curves are developed in LMM and systematic differences in groups (e.g., experimental group vs. control group) in the rate of acceleration are explored. In this paper, the intercept (initial status) as well as linear and quadratic coefficients for statistical significance and for group differences in rate of change were tested.

## RESULTS

Using schools as the units of analysis, results showed that the 20 experimental schools and 23 control schools did not differ in the banding of the schools, districts, religion, gender of the students, and source of funding. For the personal characteristics of the participants, results showed that there were no statistically significant differences between the two groups in their background sociodemographic characteristics ($p > 0.05$ in all cases), except age. In short, except that the mean age of the control group was higher than that of the experimental group, the background characteristics of the experimental schools and control schools were highly comparable at Wave 1.

The growth curve model findings based on several outcome variables are presented in Table 2. Results showed that there were significant interactions of group and waves for KEY36, CBC (cognitive-behavioral competencies, which is a second-order factor), PID (positive identity, which is a second-order factor), and academic adjustment. The interaction effects were then plotted graphically to assist the interpretation of findings. As revealed by Figs. 1–4, the findings showed that the experimental participants dropped more slowly than did the control participants. Further analyses based on experimental participants who found the program to be beneficial (responding in the direction of agreement to item 20) vs. control participants similarly showed that the experimental participants generally performed better than control participants in terms of the global positive youth development indicators (Figs. 5–8).

## DISCUSSION

The purpose of this paper is to report objective outcome evaluation findings regarding the effectiveness of a positive youth development program (Project P.A.T.H.S.) in Hong Kong using individual growth curve modeling techniques. This is the first known scientific study to adopt a randomized group trial design using data spanning over 2 years to evaluate a positive youth development program based on a curricular approach in different Chinese communities.

The findings generally showed that compared with participants in the control group, participants in the experimental schools performed better in different indicators of positive youth development. First, the findings revealed that experimental participants performed better than control participants in the areas of psychosocial competencies. For example, findings based on CBC (cognitive-behavioral competencies second-order factor) suggest that the experimental subjects displayed higher scores on cognitive competence, behavioral competence, and self-determination. Second, the experimental subjects performed better than did the control subjects in PID (positive identity second-order factor). Finally, the experimental subjects had a slower decline in school adjustment than did participants in the control schools.

Further analyses based on the experimental subjects who found the program to be beneficial to their development only (i.e., response to SOS-20 in the positive direction) showed similar, but stronger results. Besides the findings that the experimental participants performed better than did control participants in KEY15 and KEY36, the decline in overall positive youth development was slower in the experimental participants than in the control participants in CPYDS-10 (global measure of psychosocial competence and strengths, which includes resilience, social competence, emotional competence, cognitive competence, behavioral competence, moral competence, self-determination, self-efficacy, beliefs in the future, and clear and positive identity) and CPYDS-12 (all subscales, excluding behavioral competence, self-determination, and prosocial norms).

The above findings basically reinforce the previous objective outcome evaluation findings based on general linear models[7,8]. In conjunction with the previous findings based on objective outcome evaluation, subjective outcome evaluation, qualitative evaluation via focus groups, qualitative evaluation via diaries, process evaluation, and interim evaluation, the existing evaluation findings from the Project P.A.T.H.S. basically suggest that the program is an effective one. In view of the paucity of outcome studies in Hong Kong, the present study contributes to evidence-based youth work in Hong Kong[20].

Nevertheless, one interesting observation is that there was a general decline in positive youth developmental attributes across time. While this finding is consistent with the finding that adolescent mental health deteriorated across time[21], the decline in “perceived” psychosocial competence across time is an enigma deserving further investigation.

**TABLE 2**
**Growth Curve Models for Indicators Derived from the CPYDS and SA Measures**

| | Predictors | | | | | | | |
|---|---|---|---|---|---|---|---|---|
| | Subjects Joining the Tier 1 Program as Experimental Subjects | | | | Subjects Joining the Tier 1 Program and Experiencing the Program as Beneficial | | | |
| | KEY36 | CBC | PID | SA | KEY15 | KEY36 | CPYDS-10 | CPYDS-12 |
| Intercept | | | | | | | | |
| Initial status | 166.98** | 4.80** | 4.60** | 3.61** | 4.70** | 169.22** | 4.75** | 4.86** |
| Group | –0.20 | –0.03 | –0.01 | –0.02 | –0.10** | –3.24** | 0.11** | –0.10** |
| Gender | –5.74** | –0.12** | –0.06** | –0.12** | –0.15** | –5.33** | –0.14** | –0.16** |
| Age | –1.65 | –0.02** | –0.07** | –0.09** | –0.02* | –1.48** | –0.03** | –0.04** |
| Linear | | | | | | | | |
| Initial status | –17.55** | –0.33** | –0.52** | –0.69** | –0.24** | –13.23** | –0.30** | –0.37** |
| Group | –4.66** | –0.02 | –0.15* | –0.13* | –0.16** | –7.55** | –0.13** | –0.15** |
| Gender | 6.32** | 0.02 | 0.21** | 0.12* | 0.17** | 8.56** | 0.15** | 0.19** |
| Age | 2.55** | 0.03 | 0.09** | 0.12** | 0.04* | 2.14** | 0.05** | 0.05** |
| Group*Gender | 1.99 | 0.15* | 0.07 | 0.08* | 0.00 | –0.53 | 0.02 | 0.00 |
| Quadratic | | | | | | | | |
| Initial status | 7.32** | 0.14** | 0.21** | 0.22** | 0.08* | 4.69** | 0.11** | 0.12** |
| Group | 2.10* | 0.00 | 0.06 | 0.07* | 0.08** | 3.74** | 0.07** | 0.08** |
| Gender | –1.99* | 0.02 | –0.07* | –0.02* | –0.04 | –3.27** | –0.03 | –0.05* |
| Age | –1.10** | –0.01 | –0.03* | –0.05** | –0.01 | –0.81* | –0.02* | –0.02 |
| Group*Gender | –1.19 | –0.08* | –0.04 | –0.06 | –0.01 | 0.20 | –0.03 | –0.01 |

*Note*: KEY15 = indicator based on 15 key items; KEY36 = indicator based on 36 key items; CPYDS-10 = positive youth development based on 10 subscales; CPYDS-12 = positive youth development based on 12 subscales; CBC = cognitive-behavioral competencies second-order factor; PID = positive identity second-order factor; SA = school adjustment. *$p < 0.05$, **$p < 0.01$.

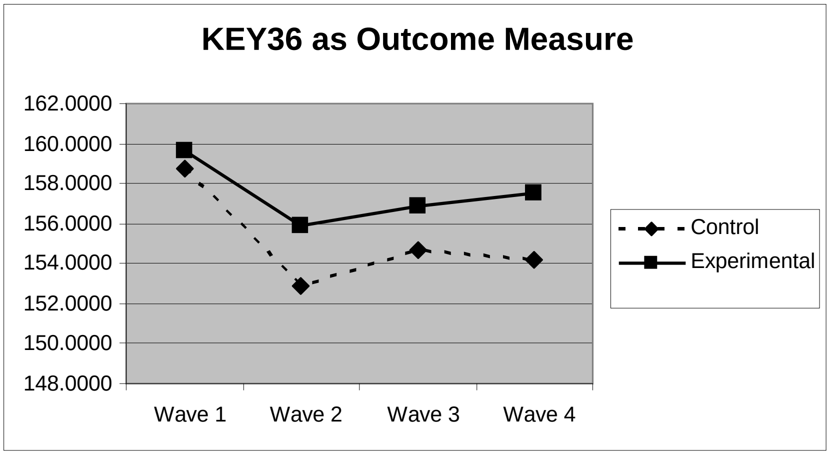

**FIGURE 1.** Growth trajectories in the experimental participants participating in the Tier 1 Program and control participants using KEY36 as an outcome indicator.

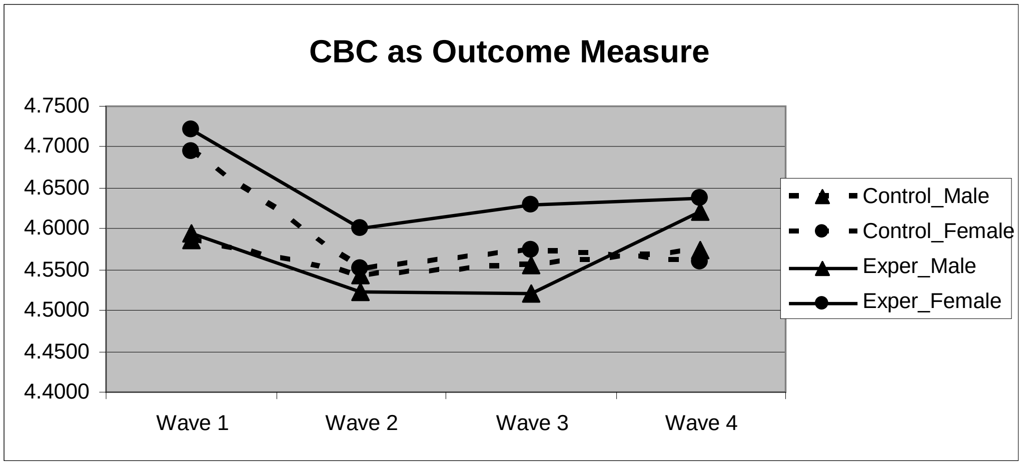

**FIGURE 2.** Growth trajectories in the experimental participants participating in the Tier 1 Program and control participants using CBC as an outcome indicator.

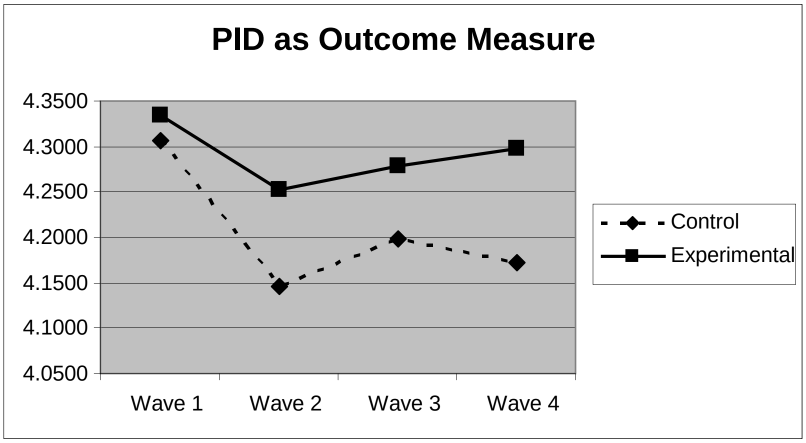

**FIGURE 3.** Growth trajectories in the experimental participants participating in the Tier 1 Program and control participants using PID as an outcome indicator.

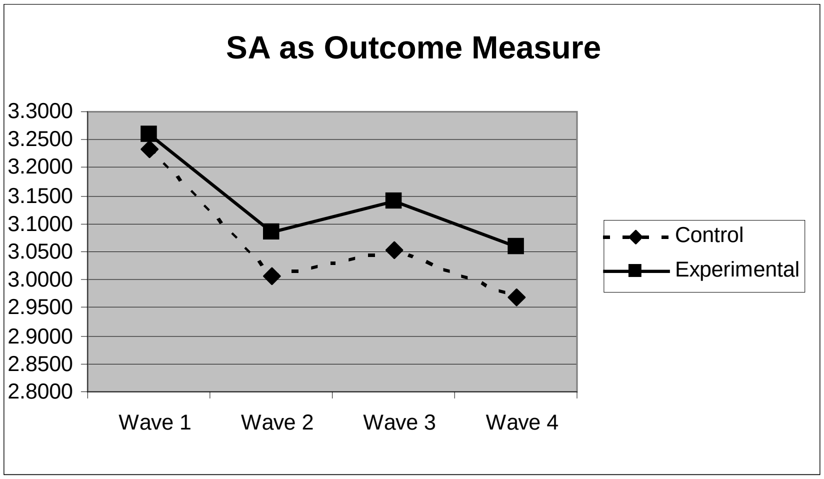

**FIGURE 4.** Growth trajectories in the experimental participants participating in the Tier 1 Program and control participants using SA as an outcome indicator.

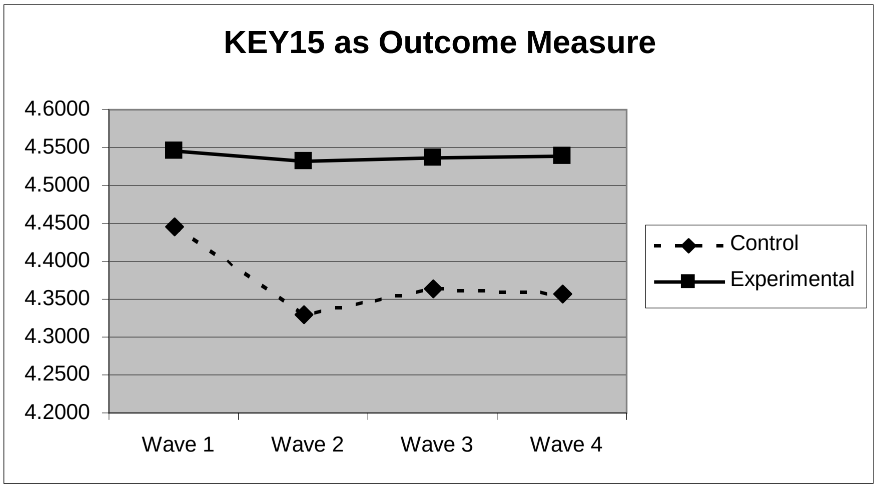

**FIGURE 5** Growth trajectories in the experimental participants participating in the Tier 1 Program (and who regarded the program as beneficial) and control participants using KEY15 as an outcome indicator.

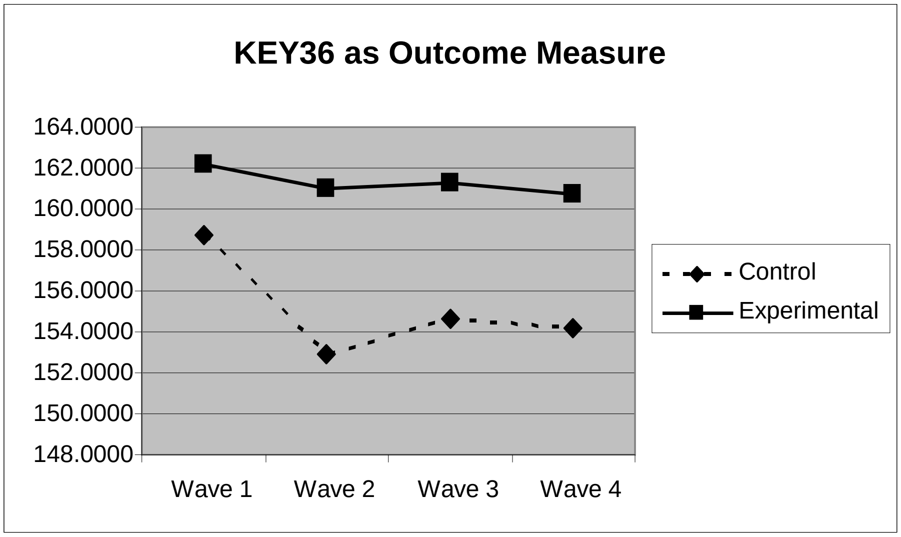

**FIGURE 6.** Growth trajectories in the experimental participants participating in the Tier 1 Program (and who regarded the program as beneficial) and control participants using KEY36 as an outcome indicator.

## ACKNOWLEDGMENTS

The preparation for this paper and the Project P.A.T.H.S. were financially supported by The Hong Kong Jockey Club Charities Trust.

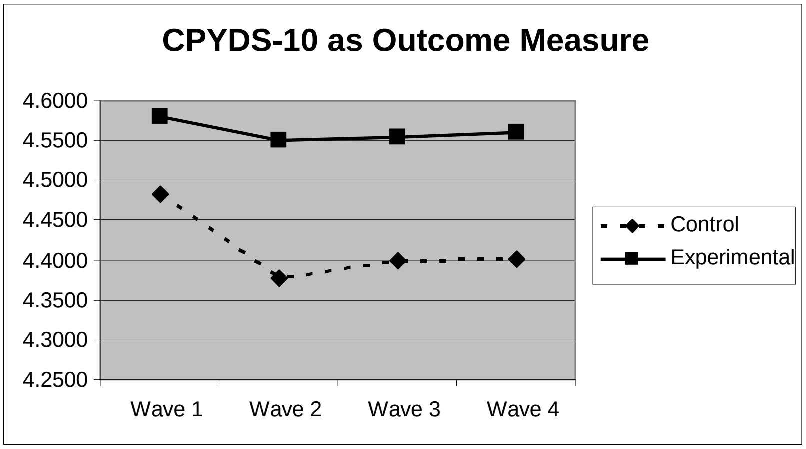

**FIGURE 7.** Growth trajectories in the experimental participants participating in the Tier 1 Program (and who regarded the program as beneficial) and control participants using CPYDS-10 as an outcome indicator.

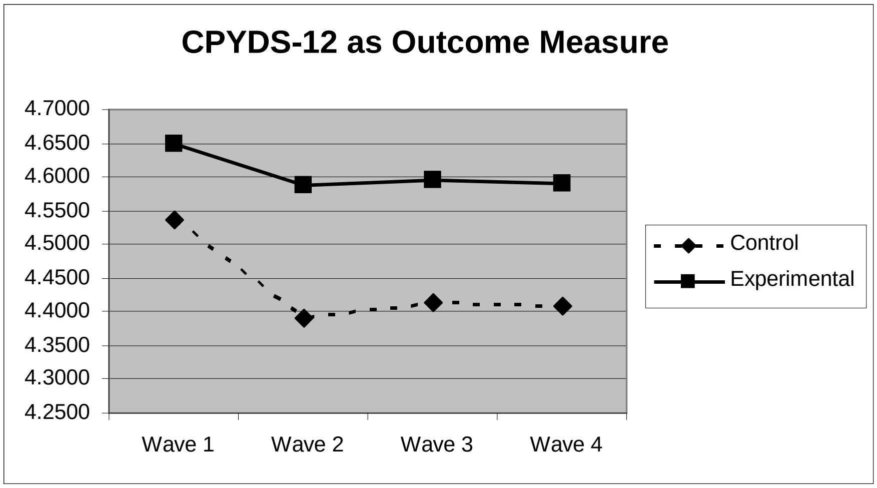

**FIGURE 8.** Growth trajectories in the experimental participants participating in the Tier 1 Program (and who regarded the program as beneficial) and control participants using CPYDS-12 as an outcome indicator.
